# Artificial Intelligence in Current Diabetes Management and Prediction

**DOI:** 10.1007/s11892-021-01423-2

**Published:** 2021-12-13

**Authors:** Akihiro Nomura, Masahiro Noguchi, Mitsuhiro Kometani, Kenji Furukawa, Takashi Yoneda

**Affiliations:** 1 Department of Biomedical Informatics, CureApp Institute, Karuizawa, Japan; 2 Innovative Clinical Research Center, Kanazawa University, 13-1 Takaramachi, Kanazawa, 9208641 Japan; 3 Department of Cardiovascular Medicine, Kanazawa University Graduate School of Medical Sciences, Kanazawa, Japan; 4 Department of Health Promotion and Medicine of the Future, Kanazawa University Graduate School of Medical Sciences, Kanazawa, Japan; 5 Health Care Center, Japan Advanced Institute of Science and Technology, Nomi, Japan

**Keywords:** Artificial intelligence, Machine learning, Diabetes, Disease prediction

## Abstract

**Purpose of Review:**

Artificial intelligence (AI) can make advanced inferences based on a large amount of data. The mainstream technologies of the AI boom in 2021 are machine learning (ML) and deep learning, which have made significant progress due to the increase in computational resources accompanied by the dramatic improvement in computer performance. In this review, we introduce AI/ML-based medical devices and prediction models regarding diabetes.

**Recent Findings:**

In the field of diabetes, several AI-/ML-based medical devices and regarding automatic retinal screening, clinical diagnosis support, and patient self-management tool have already been approved by the US Food and Drug Administration. As for new-onset diabetes prediction using ML methods, its performance is not superior to conventional risk stratification models that use statistical approaches so far.

**Summary:**

Despite the current situation, it is expected that the predictive performance of AI will soon be maximized by a large amount of organized data and abundant computational resources, which will contribute to a dramatic improvement in the accuracy of disease prediction models for diabetes.

## What Is Artificial Intelligence?

Artificial intelligence (AI) is a concept that has no single, unequivocal definition. The Japanese Society provides one example of its definition for artificial intelligence, which states, “Artificial intelligence aims to accurately make advanced inferences on a large amount of data” [1]. Since there is no unequivocal definition of the term, some AI-labeled products are not used in the AI technology we mentioned in this paper. Therefore, one must be clear about what is being referenced when the term AI is used.

Here, we briefly explain the concept of Strong AI and Weak AI [2]. *Strong AI* refers to a highly versatile AI that can establish a “consciousness” close to human thinking, make use of an appropriate program, and make comprehensive decisions. Examples to illustrate this are the Skynet from the movie The Terminator, the comic Doraemon, and C-3PO from the movie Star Wars. In contrast to Strong AI, *Weak AI* refers to the sort of AI specializing in a specific area or performs a specific task and does not have the sort of consciousness and ability to make comprehensive judgments like Strong AI. We may recognize Weak AI from news reports about the Chess computer program *Deep Blue* from IBM or the Go program *AlphaGo* from Google DeepMind beating professional human players. Self-driving technology and voice recognition technology, such as Siri on iPhone, are also examples of Weak AI. As of 2021, Strong AI is still in the research stage, and whenever we hear about the practical application of AI, this almost always refers to Weak AI. Weak AI technology is only weak in name, as some of their processing capacities no longer outperform human beings in many fields.

## How Is AI Used?

**Fig. 1:**
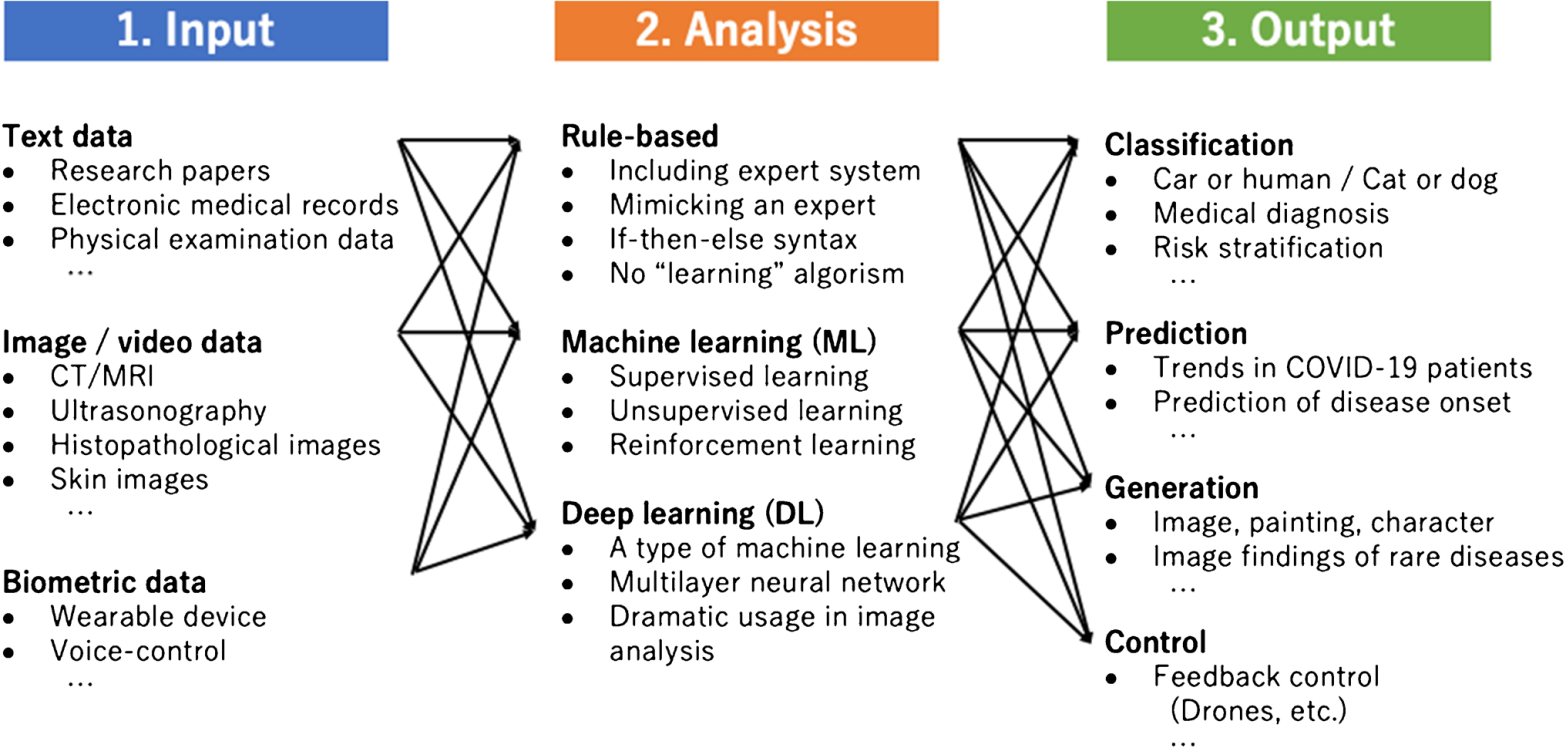
Representative flow of using AI in medicine

Figure 1 shows a representative flow of using AI in medicine. The flow is divided into three stages: input, analysis, and output. Of these, AI is incorporated in the analysis part as one of the analytical tools. In the past AI booms, rule-based algorithms such as an expert system were mainly used in medicine. However, the mainstream of the current AI boom is led by machine learning (ML) and deep learning, and the latter is a type of machine learning that has made significant progress in the last 10 years due to the increase in computational resources accompanied by the dramatic improvement in computer performance. Therefore, one must remember which kind of AI is being referenced, as AI from previous years refers to rule-based AI, whereas AI in the current medical field often refers to machine learning or deep learning. Given these circumstances, the term *Medical devices using AI* has recently been specified more clearly as *AI-/ML-based medical devices*.

Thus, the primary approach of using AI in medicine today could be to use machine learning and deep learning as analytical tools to obtain the target output. For example, if the goal is to determine whether a patient has diabetic gangrene based on skin imaging, we would develop a *classifier* using machine learning that would select images/imaging as the input, deep learning as the analytical tool, and classification purpose and output.

## Relationship Between Statistics and Machine Learning

As we mentioned, AI could be something like one of the analytical tools. Thus, one might think that AI-based analysis is just a replacement of conventional statistical methods, i.e., linear regression or logistical regression. In medicine, both *inference* and *prediction* are important goals that both conventional statistics and machine learning can achieve. However, there are differences between the approaches and goals emphasized in statistics and machine learning. Statistics emphasizes providing a framework for decision-making through *inference*. In contrast, machine learning emphasizes maximizing *predictive* performance [3•].

In statistics, we *estimate* the values in the ideal population using the data at hand based on many assumptions and perform *hypothesis testing* to assess correlations or differences between groups. When building a model, candidates for model variables (i.e., risk factors) are already determined. In other words, statistics emphasizes the process of reaching reasonable conclusions, such as the validity of statistical models, accurate estimation of each parameter, or inference from the model.

On the other hand, machine learning is used to maximize the performance of predicting answers to questions for which we do not yet know. In addition, even if the model variables are not straightforward or difficult to verbalize, it is possible to discover, generate, and select *features* that maximize the output by converting the input variables to them. In this sense, features have a similar relationship to the risk factors in the statistical processes when their importance is high, but on the contrary, they are only one component for maximizing prediction. Machine learning is sometimes compared to labeling work because it primarily learns how to judge labeling according to a specific algorithm from a large amount of input data, optimizes the model to produce better output, and then labels the new data as correct as possible. While machine learning is the same as statistics in the sense that it uses a model to produce results, to put it roughly, the goal of machine learning is achieved if the labeling prediction is ultimately best. Thus, the question of how good the model fitness is, as we see in statistics, is less important for machine learning. One of the reasons why machine learning can be regarded as a black box might derive from this unique character that the result is impressive but challenging to explain why and how it is obtained. Considering which method, statistics or machine learning, is better or worse for inference or prediction in medicine is not practical. Instead, it is better to appropriately use each technique according to what the researcher wants to obtain due to the analysis while understanding each approach’s risk, benefit, and outputs.

## AI Use in Current Diabetes Management

Next, we discuss the use of AI in medicine for diabetes, specifically in medical devices. The first AI-based medical device, BodyGuardian, was cleared by the US Food and Drug Administration (FDA) in 2012 when approval was given to a patch-like electrocardiogram equipped with an AI-based arrhythmia detection algorithm. Since then, the regulations on programmed medical devices, including AI, have advanced in various countries, including the USA, Europe, China, and Japan. Thanks to the outstanding development of deep learning technology and advancements in clinical applications these days, the number of approved AI-based medical devices has dramatically increased in both the USA and Europe in the past few years [4].

Currently, there are dozens of FDA-cleared AI-based medical devices using AI/machine learning technology. While most of these approvals are linked to radiology, cardiology, and oncology, three AI-based medical devices are related to diabetes management [5•]. In Japan, 12 types of AI-based medical devices have been approved as of 2020. However, all of them are for image analysis concerning radiology and diagnostic imaging, and there are no such medical devices approved for diabetes care.

Efforts towards the clinical application of AI in the diagnosis and treatment of diabetes are mainly categorized into four areas: (1) automatic retinal screening, (2) clinical diagnosis support, (3) patient self-management tools, and (4) risk stratification [6]. The first category is automatic retinal screening, an AI technology that automatically interprets the presence or absence of diabetic retinopathy—an important complication of diabetes—from fundus images. An example of this technology is the IDx-DR device manufactured by Digital Diagnostics Inc., approved by the FDA in 2018 for its high diagnostic performance by clinical trials [7]. Using this AI device, patients can be diagnosed with diabetic retinopathy or not without professional judgment from an ophthalmologist. Then, primary physicians can choose to have the patients with their fundus images see an ophthalmologist or re-examine the IDx-DR device 12 months later. This device facilitates the screening and diagnosis of diabetic retinopathy, especially in rural communities where patients have difficulties accessing an ophthalmologist.

The second category is clinical diagnostic support. Currently, AI technologies that mimic the “hidden tips of treatments by a specialist,” such as fine-tuning insulin dose, are being developed rather than just a support system for diabetes diagnosis itself. One example is Advisor Pro, manufactured by DreaMed Diabetes, Ltd., which the FDA approved in 2018. This system sends information obtained by continuous glucose monitoring (CGM) and self-monitoring of blood glucose (SMBG) to a cloud server and uses AI to determine and propose the necessity for insulin dose adjustments remotely. Then, physicians can review the proposals and notify patients. We introduce one of the clinical trials that evaluated the efficacy of this AI technology published in 2020 [8]. In this non-inferiority study, 108 patients with type 1 diabetes were randomly allocated to either an AI-managed group that received insulin treatments using the AI system or a manually managed group that received insulin treatments by a diabetes specialist. The results demonstrated that the targeted blood glucose concentration maintenance and hypoglycemia rates were non-inferior in the AI-guided group compared with the specialist manual managed group. In the future, there will be more situations like this where AI-based medical devices replace diabetes specialists in terms of fine-tuning insulin therapy.

The third category is the patient self-management tool. Self-management tool is familiar with some diabetes patients because they have already self-checked various biometric data such as actively measuring blood glucose levels through SMBG. With the patient self-management tools, the AI technology interprets their biometric data and alert like a diabetologist to improve the patient’s blood glucose control. The Guardian Connect System, manufactured by Medtronic, is an example of an AI system with this functionality. This system is based on CGM, has an accompanying smartphone application, and was certified by the FDA in 2018. It is characterized by using the AI to predict a hypoglycemic attack 1 h in advance based on the CGM data and alerts the patient. According to the product data, the accuracy of the alert is 98.5%, only 30 min before the onset of hypoglycemia. In this system, the AI issues alert for hypoglycemia to the patients from their biometric data, which are sometimes difficult to understand. Then, the patient can take, e.g., glucose tablets to prevent hypoglycemia and associated complications.

## Prediction of New-Onset Diabetes Using AI

Finally, the fourth category of AI usage in the diagnosis and treatment of diabetes is prediction and risk stratification. This category could be a part of preemptive medicine, accurately identifying individuals that are highly likely to develop a specific disease from the general population at the *pre-illness* stage. Thus, this technology would eventually eliminate the incidence of diabetes by implementing medical intervention for these people at a very early stage. Predicting the onset of diabetes does not happen with the advent of machine learning technology. To date, lots of diabetes onset prediction models have been created using statistics with known risk factors of diabetes in large cohorts. Abbasi et al. reported the usefulness of statistical models like logistic regression, Cox proportional hazard model, or Weibull distribution analysis to predict the onset of diabetes in non-diabetic individuals within 5 to 10 years [9]. In this report, the accuracy of prediction for new-onset diabetes within 5 to 10 years was around 0.74 to 0.94 in the C-index [9]. Despite the variance of predictive performance because of different baseline characteristics in each cohort, this result may show a relatively high level of predictive performance just by the conventional statistical models.

However, machine learning could be a promising tool that can maximize predictive performance than conventional statistics models. Table 1 demonstrates the studies predicting new-onset diabetes mellitus by machine learning models. Zou et al. [10] reported that the accuracy of new-onset DM prediction for hospitalized patients was around 0.81 using random forest. Choi et al. [11] also denoted that the area under the curve (AUC) of new-onset DM within 5 years for hospitalized patients was 0.78, but it derived from machine learning-based logistic regression. Other reports using population-based cohorts or electronic health records (EHR) denoted that new-onset DM prediction performance was around 0.84 to 0.87 in terms of AUC [12–14]. Moreover, Ravaut et al. [15] recently addressed that they could detect new-onset DM within 5 years with the performance of AUC 0.8026 using over 2 million general population with DM prevalence of just 1%. We also developed a machine learning-based prediction model to identify the diabetes signatures before the onset of diabetes using one of the machine learning algorithms, the gradient-boosting decision trees method. We recruited 509,153 annual health checkup records of 139,225 participants from 2008 to 2018 at Kanazawa city, Ishikawa, Japan. Of those, 65,505 participants without DM were included for the analysis. We identified 4,696 new-onset diabetes patients (7.2%) during the study period. Our trained model predicted the future incidence of diabetes with the area under the curve (AUC) and overall accuracy of 0.71 (95% confidence interval (CI), 0.69 to 0.72) and 94.9% (CI, 94.5–95.2), respectively [16].

**Table 1:** List of studies evaluating prediction of new-onset diabetes mellitus by machine learning models

Authors	Study population (dataset)	Target	No. of participants in dataset	% of DM in dataset	Representative ML model	Prediction accuracy	Years
Zou et al. [10]	Patients hospitalized in Luzhou, China	New-onset DM	~ 150,000	50.0%	Random forest	Accuracy: 0.8084	2018
Choi et al. [11]	Patients in Korea University Guro Hospital	New-onset T2DM within 5 years	8,454	4.8%	Logistic regression	AUC: 0.78	2019
Lai et al. [12]	Canadian Primary Care Sentinel Surveillance Network (CPCSSN)	New-onset T2DM	13,309	20.9%	Gradient boosting	AUC: 0.847 Sensitivity: 0.716	2019
Kopitar et al. [13]	Participants’ EHR data in 10 Slovenian primary healthcare institutions	New-onset T2DM by fasting plasma glucose levels	3,723	26–29%	Random forest, Gradient boosting	AUC 0.84–0.85	2020
Zhang et al. [14]	Participants in the Henan Rural Cohort Study, China	New-onset T2DM	36,652	9.2%	Gradient boosting	AUC: 0.872	2020
Nomura et al. [16]	Participants of nationwide annual checkups in Japan	New-onset DM within 1 year	65,505	7.2%	Gradient boosting	AUC: 0.71 Sensitivity: 0.422 Accuracy: 0.949	2020
Ravaut et al. [15]	Participants’ administrative health data in Ontario, Canada	New-onset T2DM within 5 years	2,137,343	~ 1%	Gradient boosting	AUC: 0.8026	2021

Abbreviations: *AUC* area under the curve, *DM* diabetes mellitus, *ML* machine learning, *T2DM* type 2 diabetes mellitus

Of those, some studies compared the prediction performance between statistical models and machine learning ones. However, at present, we cannot conclude that machine learning outperforms conventional statistical analyses for predicting new-onset diabetes from a specific population [11–14]. Moreover, an overfitting problem may occur, where the predictive accuracy is very high for a training population, but its accuracy significantly decreases for a target population. Although problems still exist using machine learning models for clinical practice to predict new-onset DM, more efficient machine learning models and more data as omics database (e.g., genomics, proteomics, metabolomics, microbiome) in addition to the above cohort datasets or electronic health records could have the potential to solve the problems and further to improve the accuracy of new-onset DM [17].

## Conclusion

AI aims to make accurate and advanced predictions for a large amount of knowledge data. As of 2021, AI most often refers to machine learning and deep learning, which have made significant progress with increased computational resources due to a dramatic improvement in computer performance. In diabetes diagnosis and treatment, AI-based medical devices have already been approved by the FDA and are available in other countries as well. Currently, many studies have used machine learning to predict the onset of diabetes. However, these machine learning approaches have not demonstrated superior performance in predicting disease onset compared to conventional statistical techniques that combine risk factors. Nevertheless, we believe that continuous research in machine learning and efforts toward its practical application will maximize the predictive performance of AI—using large amounts of organized data and abundant computational resources—and dramatically improve the predictive accuracy of disease diagnosis, prevention, and treatment in diabetes.
